# Italian Tracing System for Water Buffalo Milk and Processed Milk Products

**DOI:** 10.3390/ani11061737

**Published:** 2021-06-11

**Authors:** Giovanna Cappelli, Gabriele Di Vuolo, Oreste Gerini, Rosario Noschese, Francesca Bufano, Roberta Capacchione, Stefano Rosini, Antonio Limone, Esterina De Carlo

**Affiliations:** 1 National Reference Laboratory for Hygiene and Technology of Breeding and Buffalo Production, Istituto Zooprofilattico Sperimentale del Mezzogiorno, 84131 Salerno, Italy; giovanna.cappelli@izsmportici.it (G.C.); rosario.noschese@izsmportici.it (R.N.); francesca.bufano@izsmportici.it (F.B.); roberta.capacchione@izsmportici.it (R.C.); antolim@izsmportici.it (A.L.); direzionesanitaria@izsmportici.it (E.D.C.); 2 Ministry of Agricultural, Food and Forestry Policies, 00187 Roma, Italy; o.gerini@politicheagricole.it; 3 Agribusiness Quality Department, 00187 Roma, Italy; rosini.s@dqacertificazioni.it

**Keywords:** water buffalo, traceability milk, farmers, dairies

## Abstract

**Simple Summary:**

As buffalo milk is important in Italy, especially in the southern regions where Protected Designation of Origin (PDO) buffalo mozzarella from Campania is produced, the need has arisen to trace the path of milk from the farm to the dairy. We aimed to verify the applicability of a buffalo tracing system throughout the national territory, following the Ministerial Decree of 9 September 2014. Use of the online system is mandatory for breeders, dairies, and intermediaries, and is required to record and communicate all milk that is produced, bought, and sold. In Italy, the number of registrations is more representative in the Campania region, which has the highest concentration of dairy buffalo herds; for the other regions, the number of farms registered in the system is less representative. The system highlights that the circuit of non-PDO products absorbs 35% of the total milk produced, while 65% of the milk is processed in PDO dairies, which allows for the control of milk production throughout the territory and facilitates the control of milk that proceeds to be frozen or that can only be used in non-PDO productions; this increases the transparency of the supply chain. The system is a computerized archive of the productions for all the operators in the sector.

**Abstract:**

This document describes the development of a tracing system for the buffalo supply chain, namely an online computer system in which farmers, dairies, and brokers must maintain records of the production of milk through to the production of derivatives. The system is jointly used throughout the Italian national territory by the Istituto Zooprofilattico Sperimentale del Mezzogiorno (IZSM) and the Sistema Informativo Agricolo Nazionale Italiano (SIAN), after being made mandatory and regulated with the publication of the Ministerial Decree of 9 September 2014. Farmers are obligated to communicate their daily production of bulk milk, the number of animals milked, the number of the delivery note of the sale, and the name of the purchaser; within the first week of the month, they must communicate the milk production of each animal milked. Dairies are required to communicate the milk and the processed product (mozzarella, yogurt, etc.) purchased on a daily basis. The intermediaries are required to communicate the daily milk purchased, both fresh and frozen, the semi-finished product, and the sale of the same. The tracing system linked to the project authorized by the Ministry of Health, called “Development, validation and verification of the applicability of an IT system to be used for the management of traceability in the buffalo industry”, provides operators with the monitoring of production and sales in real time through alerts and access logs. Currently, there are 1531 registered farmers, 601 non-PDO dairies, 102 PDO dairies, 68 non-PDO intermediaries, and 17 PDO intermediaries in Italy. The system provides support for the recovery of the buffalo sector; from the analysis of the data extrapolated from the tracing system of the buffalo supply chain for the years 2016 to 2019, this paper highlights that the application of the Ministerial Decree No. 9406 of 9 September 2014 and the tracing of the supply chain have increased the price of buffalo milk at barns from EUR 1.37/kg to EUR 1.55/kg from 2016 to 2019.

## 1. Introduction

According to the most recent estimates, the world’s buffalo population amounts to around 208 million heads, mainly present in Asia (97.2%, concentrated mainly in India, China, and Pakistan); a further 1.9% are present in Africa (particularly in Egypt). In Europe, the buffalo population is estimated at 424,000 heads, and the rest are in Latin America (Brazil, Venezuela, Colombia, and Argentina) [1,2]. For zootechnical purposes, the buffalo species is now considered to have a dual purpose, although the production of milk is certainly more prevalent than meat production. In 2018, buffalo produced 127 million tons of milk around the world; in Europe, production was estimated at 390,000 tons of milk, of which 378,000 tons were produced in Italy [2,3,4]. In Italy, buffalo are mainly bred for meat production and for the production of milk to be used for dairy processing, since they are no longer used as working animals due to advances in agricultural mechanization. This occurred above all due to the foresight of buffalo breeders, who continued to believe in the potential of this animal and did not undertake the breeding of other species. Attempts to maintain the buffalo heritage in the decades following their reclamation have increased the numbers of the species to such an extent that, with its 400,000 heads, Italy raises more buffalo than any other country in Europe. From 2015 to 2019, the buffalo population in Italy increased by 7% [5,6,7].

From the analysis of the Italian buffalo heritage and management improvement incompanies, the need has arisen to trace the path of the production and origin of buffalo milk from the production at the farm to the consumer, above all because it is linked to a product appreciated all over the world, mozzarella di bufala, and other cheese products.

## 2. Materials and Methods

In the project authorized by the Ministry of Health named “Development, validation and verification of the applicability of a computer system to be used for the management of traceability in the buffalo industry”, an Italian online tracing system was developed to ascertain the traceability in buffalo rearing and the production of milk and milk derivatives. At the end of the project (30 November 2015), the validated system was implemented by the Italian Ministry of Agriculture and Forestry (MIPAAF) and the Italian Ministry of Health, which jointly promoted the enactment of specific legislation for the mandatory application of tracing. This online system is now applied nationwide and is managed by the Italian National Reference Centre on Water Buffalo Farming and Production, Hygiene and Technologies (CReNBuf) at the Istituto Zooprofilattico Sperimentale del Mezzogiorno (IZSM) and Sistema Agricolo Informativo Nazionale (SIAN) of the Ministry of Agriculture cooperative, with contributions from the Agribusiness Quality Department (DQA), which is the current control body of PDO cheese farms [8,9,10,11,12,13].

### 2.1. Buffalo Farmers

Buffalo farmers are obliged to communicate the following data to the IT platform “Traceability of the buffalo supply chain”: the daily quantities of milk produced overall by the lactating buffalo present on the farm; the workers who collected the milk; the quantities of milk produced in 24 h by each lactating buffalo present on the farm, measured as the sum of the quantities produced in the individual milkings carried out during the day; and the monthly quantity of milk produced, in 24 h, by each lactating buffalo present on the farm, which must be determined using a detection instrument approved by the Italian Breeders Association (AIA).

For those who have joined the AIA, detection data are obtained during functional checks; otherwise, the data are communicated directly by the breeder.

### 2.2. Intermediaries

Intermediaries are obliged to upload the following data to the platform:

- the daily quantities of buffalo milk and other buffalo products purchased, including in frozen form, indicating each supplier;
- the daily quantities of buffalo milk and other buffalo products sold, including frozen and forms other than fresh(freeze-dried or concentrated),identifying each recipient.

### 2.3. Dairies

The buffalo milk processors are obliged to communicate the identities of the farmers who provide the following:

- the quantities of mozzarella di bufala Campana PDO cheese produced;
- the quantities of buffalo milk mozzarella products;
- the quantities of other processed products derived from the use of buffalo milk;
- the quantities of buffalo milk and unused and possibly frozen semi-finished products.

For those who are enrolled in the Protected Designation of Origin (PDO) program, buffalo mozzarella data must be communicated on the PDO certification platform.

The data collected daily must be communicated using the IT platform no later than the second working days of the week following the detection. The quantities of buffalo milk and processed products are communicated using kilograms as the unit of measurement.

An important phase is the registration for the system, which involves the registration of the user as a legal representative on the SIAN system; the production data are recorded on the platform managed by the IZSM.

The software provides a call center service located at the Italian National Reference Center for the Management and Production, Hygiene and Technologies of the Water Buffalo (CReNBuf), which operates daily to support operators.

In case of failure to transmit the indicated data, the penalties described in the Law decree No.91/2014 of 24 June 2014 are applied [13,14].

## 3. Results and Discussion

After the application of the Ministerial Decree of 9 September 2014, as of 31December 2019, 1531 dairy buffalo farms in Italy were registered in the system, as reported in Table 1, per the Italian National Database Zootechnical Register (BDN) [5].

The Campania region has the largest number of registrations, as it has the highest concentration of dairy buffalo herds, followed by the Lazio region and then the Puglia region, which together represent the PDO area in the province of Isernia (Table 1).

For the other regions, the number of farms registered in the system is lower.

A total of 601 non-PDO dairies are registered in the system, but only 400 actually register data. There are 102 PDO dairies registered on the PDO certification portal. A total of 68 and 17 non-PDO and PDO intermediaries are registered, respectively (Table 2).

### 3.1. Farmer’s Registration

**Figure 1:**
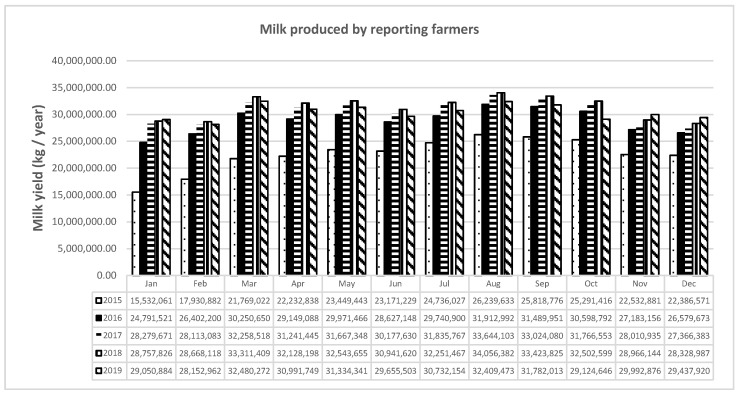
Milk produced by reporting farmers.

Between 2015 and 2019, with the application of the Ministerial Decree of 9 September 2014, users slowly began to record production data in the IT platform. By 2016, the trust acquired by breeders and the practicality of uploading data to the system had been highlighted. The functionality was confirmed by the increasing trend in registrations in2017, 2018, and 2019, which also showed an almost constant annual productivity; in 2015, apparently less milk was produced than in subsequent years (Figure 1).

### 3.2. Record Stabilization

**Figure 2:**
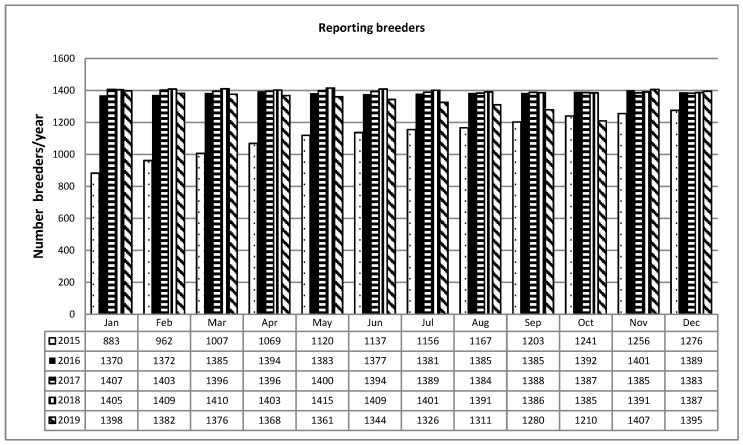
Reporting breeders.

Further confirmation of the expectations in the system is highlighted by the growing increase in the number of farmers who access and feed the database, which settled and stabilized in the years from 2016 to 2019 (Figure 2).

### 3.3. Buffalo Milked per Month

**Figure 3:**
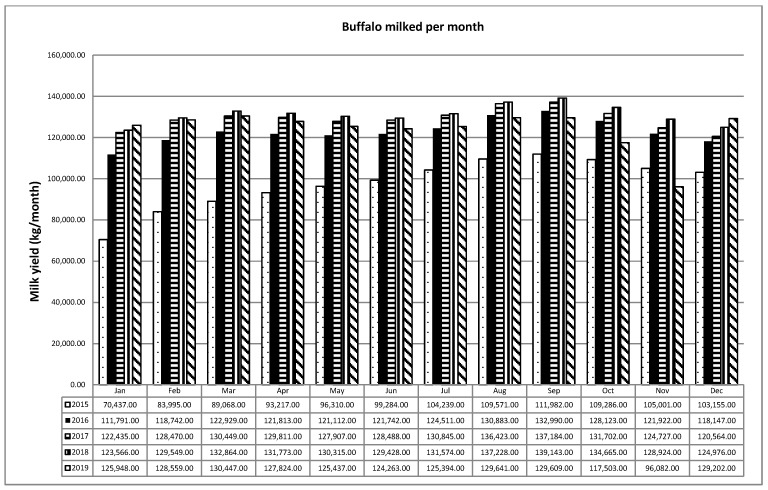
Buffalo milked per month.

Figure 3 highlights the fact that out of an Italian buffalo population of 400,000 heads, 50%are made up of nonproductive animals and the remaining 50% are made up of productive buffalo; of these, a number of animals from a minimum of “120,000 up to a maximum of 135,000” are lactating throughout the year. There is no marked seasonal adjustment of the calendar over the course of the year as the number of milking buffalo from month to month is similar, with slight increases observed in the months from August to October and from January to March. This confirms the seasonality of the animal, which, being a negative photoperiod species of equatorial origin, reproduces and gives birth in our latitude with a calendar that goes from July to March; Figure 3 highlights the incorrect application of deseasonal adjustment techniques by breeders, which would lead to a concentration of calving from January to August with a peak of animals in production from April to August, a period in which there is a greater demand for milk because there is a greater consumption of buffalo mozzarella [5,15,16,17,18,19].

### 3.4. Average Milk Production per Month

**Figure 4:**
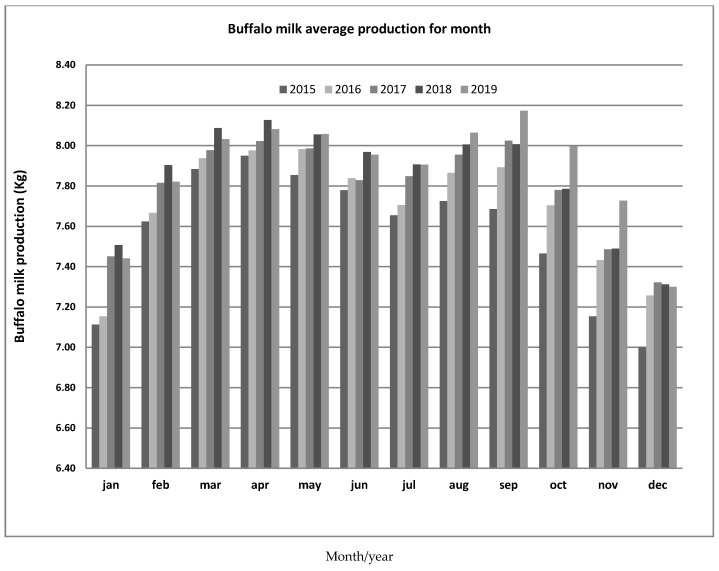
Average milk production per month.

By relating the milk produced per month over the years to the number of milking buffalo, the average production of an Italian Mediterranean buffalo can be determined: it is approximately 8kg/head/day/year, ranging from 7.70 kg in 2016 to 7.89 kg in 2019.This slight increase can be attributed to the increase in the average selling price of milk from 2016 to 2019,likely insofar as the average selling price of milk has led to a qualitative improvement in the feed ration of dairy animals; moreover, the attempt to apply a technique of seasonal adjustment of the calving calendar is evidenced by the increase in production in the following months from March in May, but the confirmation of the seasonality of the species is, instead, evident from the increase in productivity in the months from August to September; since the buffalo is a species with a negative photoperiod, it turns out to be a polyestral animal that tends to be seasonal with a short day, and therefore its productivity is accentuated in the months when the hours of light decrease (Figure 4) [15,16,17,19].

### 3.5. Non-PDO Dairies

**Figure 5:**
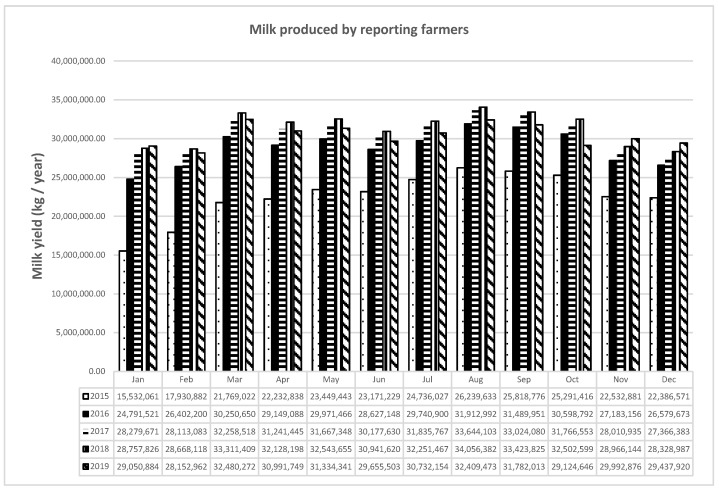
Milk processed in non-PDO dairies (kg).

**Figure 6:**
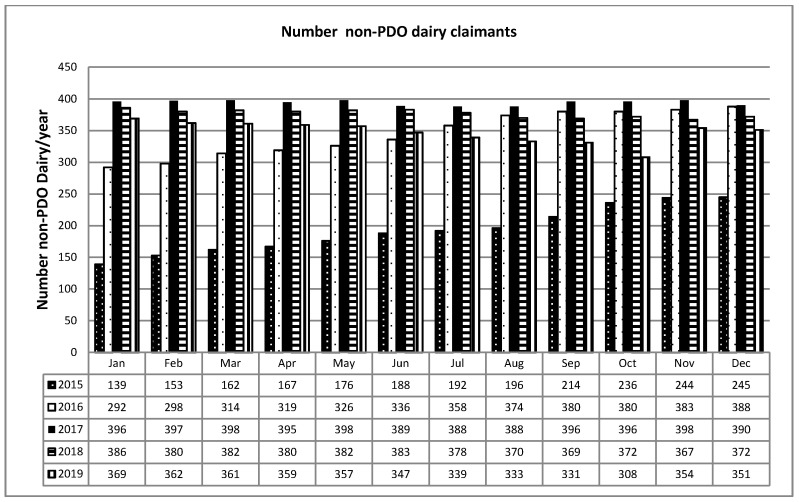
Number of non-PDO dairy reporters.

The data recording in the system showed the same trend as for breeders: in 2015, the system was initiated, the data became reliable as of 2016, and in subsequent years the trend was almost constant (Figure 5 and Figure 6).

### 3.6. Milk Purchased for Non-PDO Products

**Figure 7:**
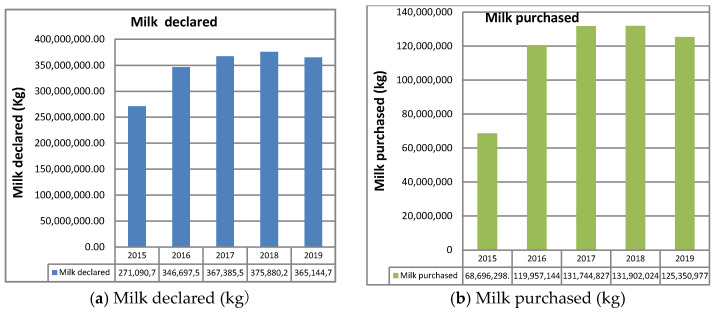
Milk product registrants (**a**) and milk purchased for non-PDO dairy products (**b**).

Figure 7 [milk product registrants (**a**) and milk purchased for non-PDO dairy products (**b**)] shows that the circuit of non-PDO products absorbed 35% of the total milk produced compared with the 65% of the milk processed in PDO dairies. The data are congruent and coherent, since (leaving out the year 2015 when the system began to be applied) 2016 to 2019 show an overlapping trend, testifying to the veracity of the data and therefore of the milk quotas absorbed by the PDO and non-PDO.

### 3.7. Milk Purchased and Milk in Non-PDO Dairy Storage

**Figure 8:**
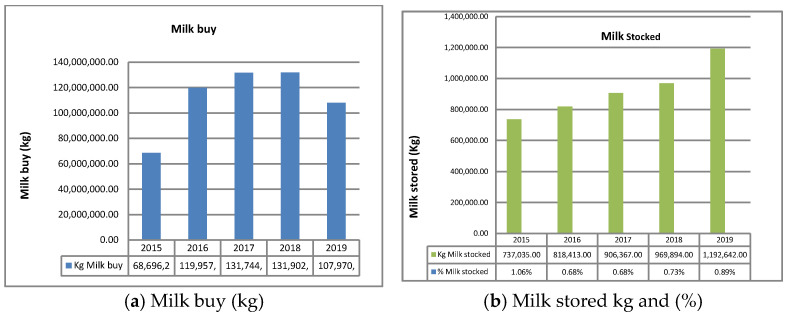
Milk bought (**a**) and milk in non-PDO dairy storage per year (**b**).

Figure 8 [Milk bought (**a**) and milk in non-PDO dairy storage per year (**b**)] compares the milk bought per year compared with the milk stored during the year. The quantity of residual milk stored at the end of the year is 0.63–0.89%, indicating that the milk bought by the non-PDO circuit is almost all processed. What appears to be residual stored milk is, in reality, the result of an imperfect clarification in the Ministerial Decree regarding the figures reported by intermediaries.

These data constitute a gap in the system; an agreement is currently being implemented between IZSM, SIAN, and DQA to ensure that the freezing plants transmit the data relating to the storage of buffalo milk at their factories in order to accurately track the batches of raw material that should be excluded from processing in the PDO chain as required by the Production Regulations. Regardless, the control bodies may consult the data in paper form, which is mandatory and available at the plants [20].

For the definition of freezing firms, monitoring of at least one year will be required.

The tracing system enables the control of the production of buffalo milk throughout the country on a weekly basis; it also facilitates control of the milk that is frozen or that can only be used in non-PDO products. This increases the transparency of the supply chain for both PDO and non-PDO products, as the control bodies can, in real time, access the system and ensure that the statements or infringements are accurate. Moreover, the system is shared with the Central Inspectorate for repression fraud-Mipaaf (ICQRF), anti-sophistication and health cores of the Carabinieri (Nas) and the AASSLL of the Campania region (Healthcare Assistance Centers), allowing warnings to be directly provided from the peripheral offices, even before the documents have been verified.

Lastly, the system provides a computer archive of the production of all food business operators, including breeders, dairies, and intermediaries.

## 4. Conclusions

To conclude, from the analysis of the data extrapolated from the tracing system for the buffalo supply chain for the years 2016 to 2019, we analyzed the application of the Ministerial Decree No.9406 of 9 September 2014. The tracing of the supply chain showed the following: the price of buffalo milk at barns increased from EUR 1.37/kg to EUR 1.55/kg from 2016 to 2019, and the non-PDO products on the market accounted for 35% of the total. This was produced by about 400 dairies, while the remaining 65% was produced by 102 dairies, highlighting the consumer preference for protected designation products over unknown ones [3,9].The lower use of frozen milk has improved the quality of the product, which has increased consumption and, consequently, sales [3].

## Figures and Tables

**Table 1:** Dairy buffalo farms registered in the traceability system in Italy.

Region	Subscribers	Farms in BDN	Percentage (%)
Campania	1060	1268	83.6
Lazio	386	547	70.6
Puglia	23	53	43.3
Molise	3	3	100
Piemonte	5	7	71.4
Liguria	0	1	0
Lombardia	9	24	37.5
Bolzano	0	1	0
Veneto	4	23	17.3
Friuli Venezia Giulia	5	5	100
Emilia Romagna	3	8	37.5
Toscana	4	5	80
Umbria	2	4	50
Marche	3	9	33.3
Abruzzo	1	6	16.7
Basilicata	11	20	55
Calabria	5	13	38.4
Sicilia	7	13	53.8
Valle D’Aosta	0	0	-
Trentino Alto Adige	0	0	-
Sardegna	0	2	0
Total	1531	2012	-

**Table 2:** Dairies and intermediaries registered in the traceability system in Italy.

Non-PDO Dairies	PDO Dairies	Non-PDO Intermediaries	PDO Intermediaries
601	102	68	17

## Data Availability

The data are not publicly available due to General Data Protection Regulation.
